# The formation mechanism of primary health care team effectiveness : a qualitative comparative analysis research

**DOI:** 10.1186/s12875-024-02278-8

**Published:** 2024-01-29

**Authors:** Chanjiao Li, Lu Cui, Siyu Zhou, Anning He, Ziling Ni

**Affiliations:** 1 Department of Health Management and Policy, School of Public Health, Hangzhou Normal University, No. 2318, Yuhangtang Rd, Hangzhou, 311121 China; 2 Department of Quality Management, Zhejiang University School of Medicine Sir Run Run Shaw Hospital, Hangzhou, 310016 China

**Keywords:** Primary health care team, Team effectiveness, Qualitative comparative analysis

## Abstract

**Background:**

Team-based care is an essential part of primary health care (PHC), and its team service delivery process is a systematic one involving multiple and complex influences. Research on the formation mechanism can help improve the effectiveness of primary health care teams (PHCTs).

**Methods:**

First, based on the Donabedian model, we explored the theoretical framework of a PHC team’s effectiveness formation mechanism. Semi-structured interviews were conducted with 23primary health care team members in Hangzhou, Zhejiang Province, China. A total of seven factors were then included as conditional variables using the crisp set qualitative comparative analysis (csQCA) to explore the complex influences between them and the outcome variable through univariate necessity analysis and path configuration analysis.

**Results:**

Univariate necessity analysis showed that only “Clear Goals” in the structural dimension were necessary for team effectiveness perception. Six pathways to good primary health care team perception of effectiveness were identified. Two of these paths were more typical.

**Conclusion:**

“Clear Goals” was the core variable that should be emphasized when exploring the mechanism of PHCT formation. The results suggest that human resources in the management team should be rationally allocated, goal-oriented, and given good attention. Future studies should explore complex combinations of PHCT factors to improve the effectiveness of PHCTs.

**Supplementary Information:**

The online version contains supplementary material available at 10.1186/s12875-024-02278-8.

## Introduction

Chronic diseases such as cardiovascular diseases, cancer, diabetes, and respiratory diseases are currently the most important public health problems worldwide [1]. Due to changes in the living environment and increased life stress, the incidence of chronic diseases is increasing annually; most patients present with multiple chronic diseases, and this group of patients is becoming younger and more common [2]. Existing changes have made it difficult for the traditional model of health care services to meet residents’ health needs. To effectively manage the health of residents and improve hierarchical medical systems, a primary health care (PHC) service model based on general practice teams has been established by relying on PHC institutions.

Pregnant women, children, the elderly and people with chronic diseases are key populations in need of primary health care services [3]. PHCT collaboration also benefits patients with multiple chronic illnesses [4]. By including other health care professionals, patients will have access to more diverse services [5]. A primary health care team (PHCT) is composed of a general practitioner who has core responsibility, nurses as the main support staff, and other personnel such as public health physicians, pharmacists, nurse assistants, and community volunteers who play an auxiliary support role [6]. This team-based care (TBC) model allows team members to work together around a common goal and share the responsibility for achieving their mission. Therefore, TBC, through inter-professional collaboration to achieve common goals, is encouraged and meaningful.

However, there are many practical problems in providing team-based health care services. Medical errors can occur if critical information is not passed on, the information is misinterpreted, or the next steps are unclear in a team. Lack of role clarity can create chaos in the team, resulting in suboptimal care for patients [6]. In addition, there is a lack of clear team goals; the focus is only on performance targets issued by management. The team communication mode is mainly top-down without establishing a horizontal communication mode with wide participation. The relationship between team members is based on information sharing and a lack of effective synergy [7]. These issues put the effectiveness of the PHCT at risk and are directly related to the quality of health care service delivery.

Consequently, it would be meaningful to examine the factors that influence the effectiveness of PHCTs or strategies to enhance their effectiveness. Previous research has focused on analyzing factors that influence the effectiveness of PHC services from the patient's perspective [8–10], as well as using quantitative or qualitative research methods to explore the influencing mechanisms of multiple factors [11–15]. Despite progress in the above studies, some issues remain. Team effectiveness can be perceived differently depending on the viewpoint. Patients may estimate a team’s effectiveness based on the services received, whereas team members may focus more on job satisfaction and achieving shared team objectives [16, 17]. Most current studies have been conducted from the perspective of service utilizers, emphasizing how to maximize benefits to patients. However, because the PHC service process is a complex systemic process involving both service providers and users, it is extremely difficult to obtain a complete and comprehensive analysis from the perspective of a single party. Neglecting the interests and behaviors of service providers cannot solve the problem of their low effectiveness [18]. However, existing research on the effectiveness of PHCTs lacks a comprehensive investigation from the perspective of service providers. Therefore, to avoid nonsystematic analyses arising from a single perspective, studies based on service providers’ experiences and perceptions should be included to provide more robust evidence. In addition, in terms of research methodology, the results of relevant quantitative studies on the effectiveness of PHCTs are based on data analysis [7, 19, 20], which is unable to test the deeper reasons behind the emergence of behaviors. In contrast, purely qualitative research methods focus on exploring a single path to solving problems, but reveal their shortcomings when addressing systemic problems with multiple complexities. Hence, effectively combining both quantitative and qualitative methods can be powerful.

The emergence of PHCT effectiveness as a complex sociological phenomenon with diverse and complex causes leading to different outcomes is not the result of a single factor [7]. Unlike the traditional case and regression analysis, which are the classical methods in health management, qualitative comparative analysis (QCA) is a case study-oriented theoretical collection research method between qualitative analysis and quantitative analysis. For a list of terms used in QCA, see Table 1. QCA analysis is based on the idea of set theory to analyze the relationship between the condition set and the result set of the case. The principle is to conceptualize cause conditions and outcome variables into sets, and then reveals complex causality by analyzing the adequacy and necessity of conditions or combinations of conditions for results [21]. Therefore, we introduced a QCA method that combines quantitative research and case studies from a set theory perspective [22]. The quantification of data based on an in-depth understanding of the cases is an attempt to explore the combination of causes that impact the ending variables. QCA can be used to address the causal complexity of health systems [23, 24]. Our study applied the QCA method to the research field of PHCT effectiveness to provide new perspectives for future research on complex health management issues.

**Table 1:** Key terms in qualitative comparative analysis (QCA)

Key term	Definition
Necessity	A condition is necessary if it is always present when the outcome is present
Raw coverage	Percentage of total cases covered by a path (number of cases divided by the total number of cases)
Unique coverage	Percentage of the total cases covered only in this path (number of unique cases divided by the total number of cases)
Solution coverage	The extent to which all combinations of conditions cover the cases
Truth table	All the condition combinations empirically found
Consistency	Degree of association between the conditional combinations of the results of truth table operations and the realistic combinations of the sample cases
Crisp set QCA (csQCA)	This form of QCA allows only binary forms of conditions. In set theory terms, conditions fall in (labelled as 1) or out (labelled as 0) of the sets
Solution	All the paths that result from the analysis. There are three types of solution: complex, intermediate and parsimonious
Complex solution	No assumptions are made about the logical remainders in this solution
Intermediate solution	Uses a combination of both theory and the empirical cases to determine the paths. The empirical paths will never be contradicted in this solution
Parsimonious solution	The most simple solution that uses mainly theory with the empirical cases to derive the path solutions

Consequently, our study takes the service experience and work perception of service providers in meeting the complex treatment needs of patients as the entry point, and the theoretical framework of the formation mechanism affecting PHCT service efficiency is extracted based on Donabedian's three-dimensional model. The crisp set qualitative comparative analysis (csQCA) used in this study is an analytical technique. The truth table is established by binary assignment of the condition variable and the result variable, and then the necessity analysis of a single variable is carried out. Finally, the influence of different combinations of condition variables on the result variable is studied by conditional combination analysis. This is used to explore the combination of multiple complex factors that affect the effectiveness of PHCT services. The purpose of this study is to investigate the causal pathways to enhance the level of services provided by PHCTs, provide new suggestions to improve the service capability of PHCTs, and establish a harmonious doctor-patient relationship.

## Methods

### Participants

Our research site selected Hangzhou, Zhejiang Province, China. First, we selected one community health service center in four central urban areas (West Lake, Shangcheng, Binjiang, Gongshu) and tow remote urban areas (Fuyang, Tonglu). Then, two family physician teams with better and worse performance were selected in each community health service center based on performance each. Finally, 23 team members were selected from the 12 teams mentioned above for interviews. Inclusion criteria for interviewees were as follows: (i) PHCT members, (ii) > 5 years of contracting experience, and (iii) interest in this study and willingness to participate in the interview process. The number of interviewees satisfies the principle of information saturation [25]. The internal conditions of these teams are diverse. The differences in teamwork content and performance appraisal methods were relatively small, but there were large variations in team structure, organizational background, team size, and other basic conditions within the team. This differentiation is helpful for a better comparative analysis of the formation mechanism of PHCT effectiveness.

### Theoretical framework of PHCT effectiveness formation mechanism

The Donabedian Model is a health care evaluation framework with a three-dimensional categorical structure that encompasses three major aspects of health care quality: the structure, process, and outcome [26].

The Donabedian model opens a new perspective for evaluating the quality of medical services in three dimensions: Structure, Process, and Outcome. The objectivity and practicality of its evaluation are greatly enhanced because of its flexible structure, focusing on assessing the service quality of medical institutions, and the fact that long-term results need not be considered [27]. For the PHCT, the quality of team service is an important element. Therefore, this study used the Donabedian model in an empirical and perceptual study of PHCT effectiveness based on a thorough comparison of common evaluation frameworks in the health field.

**Fig. 1:**
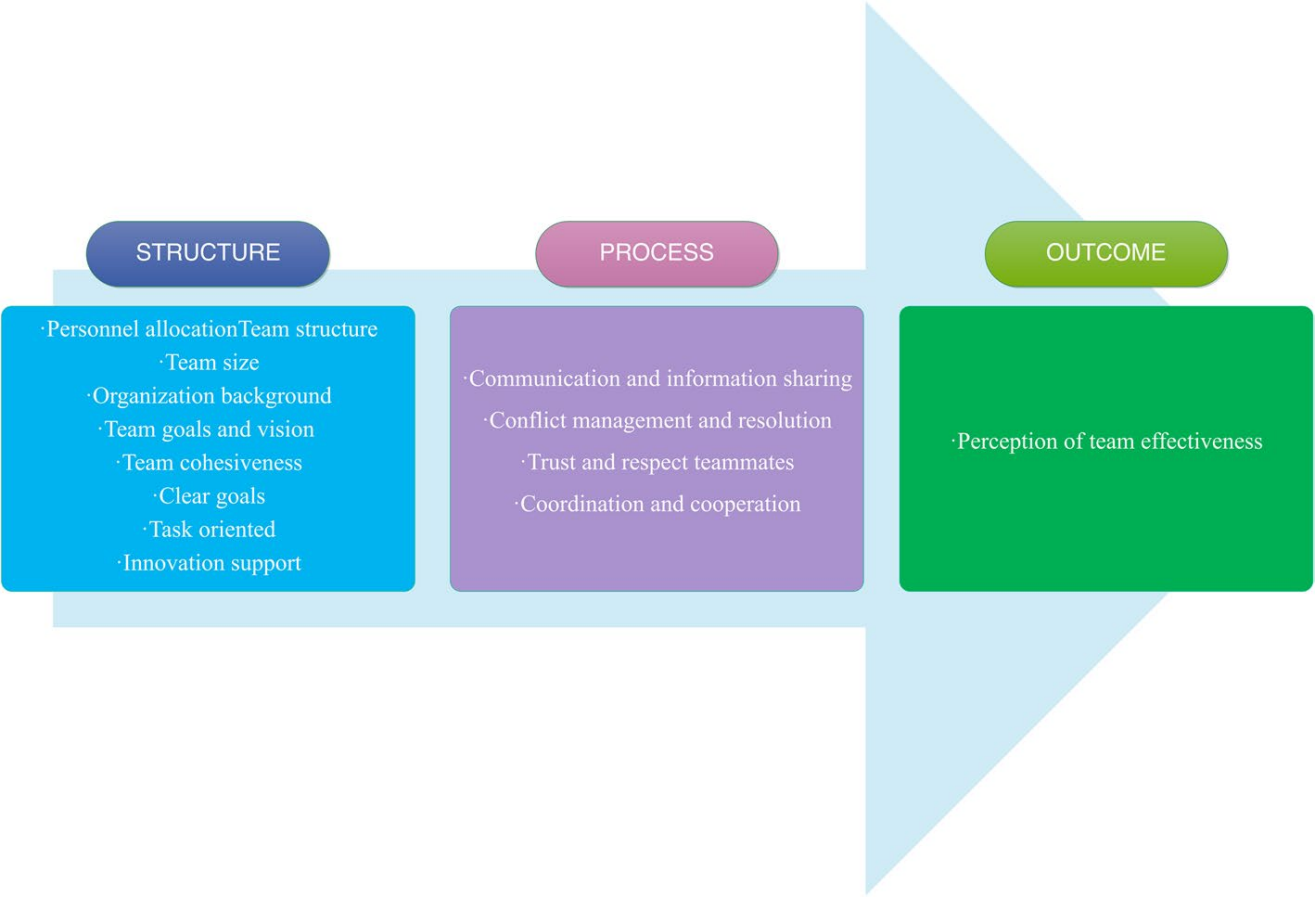
Theoretical framework of PHCT effectiveness formation mechanism

We found 13 themes in our knowledge review of the previous literature. Then, we categorize the topics under the framework of Donabedian model. Finally, a theoretical framework suitable for this study is obtained, as shown in Fig. 1. The structural dimension is the basis, the process dimension is mainly combined with practice on the basis of the structural dimension, and the result dimension is the final expression of the two, which is reflected in the effectiveness of PHCT in this study.

### Semi-structured interview

Semi-structured interviews with PHCT members provided us with the main data to analyze the responses to the research questions. An interview guide consisting of open-ended interview questions was constructed under the guidance of the Donabedian model (See Additional File 1 for an outline of the interview). The interviews covered basic information on providing services in the form of a team, the promotional and obstacle factors encountered in the process of team service, and the support conditions they expected. The interviews were pilot tested with two members of the PHCT from the community of the Tianshui Wulin Street Health Service Centre in Xiacheng District, which led to minor changes in the interview outline. Two researchers (CL and LC) conducted systematic interviews and qualitative analyses from January to May 2022 after internal training. Team members were briefed on the content and objectives of the study either face-to-face or by telephone. Consent was obtained from all participants. No other private or work relationships existed between the interviewees and interviewers prior to the start of the study. The formal interviews were conducted one-on-one and lasted approximately 20 min. The interviews were properly recorded. Within 24 h after the interviews were completed, two researchers worked together to compile the interview data (CL and AH). After integrating the recordings and transcripts and manually converting them into transcripts, we sent the interview transcripts back to the interviewees for verification and validation to obtain the final interview data.

### Analysis

We used the qualitative analysis tool NVivo11 [28] to code the interview transcripts. Two researchers (CL and AH) coded the interview content based on the interview records and read it sentence by sentence. All interviews were re-read and checked (SZ), resulting in minor adjustments to individual themes.

QCA was used for an in-depth analysis of interview results. QCA is a hybrid research method with both a case study orientation and quantitative analysis. There are two common procedures for conducting a QCA: crisp and fuzzy sets. In our exploration of the effectiveness of the PHCT, a crisp set was chosen because it allows for the most interpretable results and clarifies the resulting paths clearer [21]. The basic idea of csQCA is to specify the social phenomenon to be analyzed as relatively clear ending variables, such as whether a specific event occurs or whether a specific effect occurs, and then transform its potential influences into a number of dichotomous conditional variables (0 = absent, 1 = present) to construct a truth table [22]. Then, necessity analysis was performed for the univariate analysis in the truth table. When the sample cases passed the necessity analysis, an independent variable grouping analysis was performed. We used consistency and coverage for parameter control and ultimately analyzed the combination of key factors that had the most explanatory power.

### Data protection and ethics

Participants provided their consent for participation at the beginning of each interview. Participation in the study was voluntary and no financial compensation was received.

## Results

### Variable selection and assignment

According to the extensibility between the number of cases and antecedent conditions, the number of conditional variables should be four to seven for a medium-sized sample (10 to 40) in this study [29]. Based on the analysis of previous studies [7, 17, 30–33], this study formed interviewees’ opinions on factors related to the formation of PHCT effectiveness based on the results of qualitative interviews with PHCT members under the guidance of the Donabedian model “Structure-Process-Outcome” framework (Table 2). We organized the variables in Table 2 according to frequency, and selected the top 7 of the most frequently mentioned variables as the condition variables for this study. They are: “Personnel Allocation (PA),” “Team Structure (TS),” “Team Cohesiveness (TC),” “Clear Goals (CG),” which were mentioned most frequently in the structural dimensions during the interviews; and “Coordination and Cooperation (CC),” “Conflict Management and Resolution (CMR),” “Communication and Information Sharing (CIS)”. The above seven factors were used as conditional variables. The “Perception of Team Effectiveness (PTE)” in the outcome dimension was used as the outcome variable and each factor was assigned a value according to the assignment criteria. Table 3 (be placed at the end of the document text file) summarizes the results of variable assignments.

**Table 2:** Interviewees' perceptions of PHCT effectiveness formation

Dimension	Factors	Definition	Samples of statements	Number of times mentioned
1. Structural	1a. Clear Goals (CG)	Have a clear understanding of your own work and identify with the clear goals of the team	“We have three team members, one is responsible for diabetes and two are responsible for hypertension. This is how we divide the division.”	22
	1b. Team Structure (TS)	The team is made up of people with different backgrounds, skills, and knowledge	“In our team we have a doctor, a pharmacist, a rehabilitation therapist, a dietitian and two nurses.”	17
	1c. Personnel Allocation (PA)	To ensure the proper functioning of the organization's activities by providing the appropriate personnel to fill the various posts provided for in the organization's structure	“The team makes a two-way selection every year. In order to ensure the efficient operation of the team, we will not select you in the second year if you did not do well in the previous year.”	14
	1d. Team Cohesiveness (TC)	The attraction of the team to its members, the centripetal force of the members towards the team, and the mutual attraction of the team members to each other	“I think the head nurse in my team works very well with me.Basically, except for the signing (patients), I am in charge of the quantity, and she is in charge of the quality of the following follow-up. She is very meticulous in doing things.So this is equivalent to me is extensive, she is careful, just be complementary.”	6
	1e. Group Size (GS)	The number of people an organization has, and the relationship between the interactions of those people	“I think the strength of one person is limited, and it is necessary to rely on teamwork. I also have other responsibilities, so there will be some overtime.”	3
	1f. Task-Oriented (TO)	A collective term that refers to behaviors that guide the task. It emphasizes the guiding and regulating role of the task	“At present, I think the main work of the team is still completed according to the goals of the superiors. At the beginning of every year, the leader will give us a target time node.”	3
	1 g. Team Targets and Visions (TTV)	The expectation of the future development of the organization and individuals in an uncertain and unstable environment, which will guide or influence the actions and behaviors of the organization and its members	“The future development direction of a good team should be to maximize the function of the team as a studio. The future direction is to integrate these resources.”	3
	1 h. Innovation Support (IS)	The team can provide support for the cultivation of team members' innovative ability and creative thinking training	“I hope to organize further training for us to participate in, so that we can grow and improve our work.”	2
	1i. Background of the Organization (BO)	The organization supports (or hinder) the development of the team	“We want the information software to be optimized. For the management of diabetes, follow-up information cannot fully reflect the dynamic progression of the patient's disease, and more scientific software may be needed to conduct fine management of patient information.” “Supervision and assessment methods need to be more flexible. For example, patients with chronic diseases need four face-to-face visits, and some people live far away or are inconvenient, so we use telephone follow-up. However, the assessment does not count our face-to-face follow-up, which is considered unqualified. Therefore, the assessment needs to be humanized, otherwise it will increase our work burden.”	2
2. Process	2a. Coordination and Cooperation (CC)	Work in different positions to the best of your ability and coordinate with other members to maximize team efficiency	“Some nurses in the team have a better ability to give health education lectures, which is her advantage. Each post is actually playing its maximum capacity.”	13
	2b. Communication and Information Sharing (CIS)	The process of transferring ideas and feedback between team members, sharing patient information with others on the team	“The cooperation within our team is quite good, and members also communicate with each other in a timely manner according to the situation of patients.”	8
	2c. Conflict Management and Resolution (CMR)	Manage conflict with certain interventions to maximize its benefits and curb its harms	“For example, when our team needs to complete a certain task, there will be conflicts due to different understanding between team members when conveying information.” “There will be measures such as performance appraisal and fines for members' completion of work.”	4
	2d. Trust and Respect Teammates (TRT)	Believe in the skills and abilities of team members that will help the team succeed and show them respect	“The internal relationship of our team members is relatively harmonious. Team members have the ability to provide targeted services to patients.”	3

**Table 3:** Variable valuation table

Variable Type	Variable	Secondary Variables	Measurement
Conditional Variables	Structural Dimension	Personnel Allocation (PA)	If sufficient manpower is available in my team to provide complete PHC services, the value of 1 is given, otherwise 0
		Team Structure (TS)	If members in my team have the service ability and skills needed by the team and have different academic backgrounds, they can provide diverse perspectives and experience for team work, the value of 1 is given, otherwise 0
		Team Cohesiveness (TC)	If members in my team have similar values and it was clear that they were a team, the value of 1 is given, otherwise 0
		Clear Goals (CG)	If members in my team have a clear and unambiguous understanding of the division of labor between themselves and others, the value of 1 is given, otherwise 0
	Process Dimension	Coordination and Cooperation (CC)	If members in my team actively seek cooperation among themselves and can be provided with useful ideas and practical help from other members, the value of 1 is given, otherwise 0
		Conflict Management and Resolution (CMR)	If the team I work for faces up to the differences among members and tries to solve them, the team has a sound conflict management mechanism, the value of 1 is given, otherwise 0
		Communication and Information Sharing (CIS)	If members in my team can carry out good communication and mutual understanding, they can obtain accurate information about patients from other members, and will actively provide information about patients to other team members, the value of 1 is given, otherwise 0
Result Variables	Outcome Dimension	Perception of Team Effectiveness (PTE)	If we can always achieve our team goals and I believe that our team is efficient and the quality of our service is high, the value of 1 is given, otherwise 0

### Establish the Truth Table

According to the previous assignment criteria for the conditional and result variables, we input the raw data binary table after the dichotomous assignment into the QCA3.0 software [34] to perform the operation and build up the truth table as shown in Table 4.

**Table 4:** Crisp set truth table for Conditional and Result variables

PA	TS	TC	CG	CC	CMR	CIS	Number
0	1	0	1	1	0	0	3
0	1	0	1	0	0	0	2
1	1	0	1	1	0	0	2
1	1	1	1	1	0	0	2
0	0	0	1	0	0	0	1
1	0	0	1	0	0	0	1
1	1	0	1	0	0	0	1
1	1	1	1	0	0	0	1
0	0	0	1	1	0	0	1
1	1	0	1	0	1	0	1
1	0	0	1	0	0	1	1
1	1	0	1	0	0	1	1
1	1	1	1	0	0	1	1
1	1	0	1	1	0	1	1
1	1	1	1	1	0	1	1
0	0	0	0	1	1	1	1
1	0	0	1	1	1	1	1
0	1	1	1	1	1	1	1

0 = absent; 1 = present; *PA*  Personnel Allocation, *TS*  Team Structure, *TC* Team Cohesiveness, *CG*  Clear Goals, *CC*  Coordination and Cooperation, *CMR*  Conflict Management and Resolution, *CIS*  Communication and Information Sharing

### Univariate necessity analysis

The necessity analysis allowed us to explore the extent to which a single variable among the selected variables explained the outcome. If a certain condition always exists when a result appears, we consider it to be a necessary condition for the existence of the result. In conventional QCA operations, univariate necessity analysis is primarily determined by the consistency index. The consistency index mainly measures the degree of correlation between conditional and outcome variables. In other words, it measures the explanatory power of a combination of conditional variables for the outcome variable. For QCA, the consistency index for set-theoretic relationships is equivalent to the p-value in conventional statistical analysis. A consistency greater than 0.90 indicates a strong empirically significant set relationship, just as a p-value less than 0.05 indicates a low probability that the outcome in a traditional statistical analysis is a chance observation. The formula can be simplified as follows:

$$Consistency\;\left(Yi\leq Xi\right)=\sum\left(min\left(Xi\leq Yi\right)\right)/\sum\left(Yi\right)$$

*Xi* refers to the affiliation score in the combination of conditions, and *Yi* refers to the affiliation score in the results [35]. In general, the results can be considered necessary only if the consistency is greater than 0.9 [36]. Furthermore, the coverage index can be used to determine the strength of the explanation of the condition of the results [29]. Coverage indicates the explanatory power of the combination of conditions of the results. The closer it is to 1, the stronger the explanatory power. The results of the univariate necessity analysis using csQCA are shown in Table 5. For univariate necessity, only the “Clear Goals (CG)” dimension constitutes a necessary condition for team effectiveness perception (Consistency = 0.9545450.9). Here, it is vital to emphasize that, although the above necessary conditions necessarily exist in cases where the outcome variable takes the value of 1, cases that meet the above necessary conditions do not necessarily result in perceived team effectiveness. Thus, the necessary conditions cannot be considered sufficient. The complex combination of factors that drive high PHCT effectiveness must be extracted through the next step of multifactorial configuration analysis.

**Table 5:** Univariate necessity analysis

Variable Name	Consistency	Coverage
PA	0.590909	-
TS	0.772727	-
TC	0.272727	-
CG	0.954545	0.954545
CC	0.590909	-
CMR	0.181818	-
CIS	0.363636	-

Only when the consistency reaches 0.9 or more and the necessary condition is satisfied, the coverage rate needs to be calculated; *PA* Personnel Allocation, *TS* Team Structure, *TC* Team Cohesiveness, *CG* Clear Goals, *CC* Coordination and Cooperation, *CMR* Conflict Management and Resolution, *CIS* Communication and Information Sharing.

### Path configuration and analysis

According to the analytical principles of QCA, the necessary conditions are no longer included in the path configuration and analysis. We only analyzed other variables, thus studying the effect of different combinations of conditional variables on the outcome variable.

The complex solution, intermediate solution, and parsimonious solution were obtained by Boolean algebra (setting the threshold of Raw to 0.8; PRI value to 0.75). Referring to current mainstream research on QCA methods, most sociologists agree that intermediate solution that are reasonably well founded, moderately complex, and do not allow for the elimination of necessary conditions are the preferred choice for reporting and interpretation in QCA research [37]. Therefore, this study focused on explaining the connotations of intermediate solutions. We have translated the results of the conditional combination analysis operation of the intermediate solution (see Additional File 2) and presented them in Table 6.

**Table 6:** Generate path solutions(based on intermediate solution)

Path number	Conditional combination	Raw coverage	Unique coverage	Consistency
A	PA*TS* ~ CMR	0.454545	0.363636	1
B	~ PA* ~ TC* ~ CMR* ~ CIS	0.318182	0.318182	1
C	PA*TS* ~ TC* ~ CC* ~ CIS	0.0909091	0.0454546	1
D	PA* ~ TC* ~ CC* ~ CMR*CIS	0.0909091	0.0454546	1
E	~ TS* ~ TC*CC*CMR*CIS	0.0909091	0.0909091	1
F	~ PA*TS*TC*CC*CMR*CIS	0.0454545	0.0454546	1
Solution coverage: 1				
Solution consistency: 1				

*PA* Personnel Allocation, *TS* Team Structure, *TC* Team Cohesiveness, *CG* Clear Goals, *CC* Coordination and Cooperation, *CMR* Conflict Management and Resolution, *CIS* Communication and Information Sharing

^*^means " and "; ~ means " no"

The results of the path configuration analysis in Table 6 show that six different combinations of conditions reached the outcome of the perceived achievement of PHCT effectiveness in 23 cases. The solution coverage and consistency of the intermediate solution was 1, which proves that it has strong explanatory power for the 23 selected cases. The consistency index for all antecedent conditional constructs was 1 (> theoretical value 0.8), indicating that all cases in the six antecedent conditional combinations satisfied the consistency condition; that is, all six antecedent conditional combinations were sufficient conditions for the perceived effectiveness of the PHCT. It is easy to see that “PA” appears in all three combination paths except for the necessary condition “CG,” which is consistent with the result that the consistency of “PA” is second only to the necessary condition “CG” in the necessity analysis. Further observation of the six-cause combinations revealed that two-cause combinations were more typical than the others, with raw coverage higher than 10% [38]. In accordance with the principles of QCA, we combined the variable “Clear Goals (CG)” from the previous necessity analysis with the above combination of conditions to obtain the following two typical combinations of reasons: (A) Personnel Allocation*Team Structure*Clear Goals* ~ Conflict Management and Resolution. This explanatory pathway suggests that when teams are adequately staffed with team members from different disciplinary backgrounds and have clear and well-defined team goals, PHCTs can be effective at the desired high level even without conflict management and resolution mechanisms. (B) ~ Personnel Allocation* ~ Team Cohesiveness*Clear Goals* ~ Conflict Management and Resolution* ~ Communication and Information Sharing. This explanatory pathway “necessarily excludes” elements of personnel allocation, team cohesiveness, clear goals, conflict management and resolution, communication, and information sharing, highlighting the direct impact of the “Clear Goals” element in producing high performance in PHCTs.

## Discussion

This study complements previous studies related to PHCT effectiveness from the perspective of service providers. We assessed 23 PHCT members’ perceptions of team-based service effectiveness. Based on the perspective of service provider experience and perception, semi-structured Interviews were used to explore the factors related to team efficacy that PHCT teams face in delivering services as a team. Following exploration, the interview data were further processed to build a framework for the factors influencing the effectiveness of PHCT services. The cases were then quantified using the QCA method to quantify the data and explore the pathways of conditional factor combinations with explanatory power to fill methodological gaps. Our findings suggest that PHCT service effectiveness is influenced by a combination of multiple factors that constitute multiple complex combinations of conditions and thus are influenced to produce change.

In this study, we found that having clear goals for teamwork was an important facilitator of the effectiveness of PHCT in delivering services as a team, which was affirmed by team members in the interviews. Among the results obtained from the univariate necessity analysis, only “Clear Goals (CG)” constituted a necessary condition for the generation of the outcome variable (Consistency = 0.9545450.9). In other words, the presence of a conditional variable for clear goals is necessary for the perceived effectiveness of the PHCT. Previous research suggests that without the central role of clear team goals, it is difficult to achieve high PHCT performance [39]. Team goals are organization-oriented, and managers and team members work together to establish clear team goals so that individual and team goals can be aligned to the greatest extent possible. Research has shown that team processes such as clear goals and objectives are among the main factors influencing interdisciplinary teamwork [40]. The team members have a clear division of labor and work closely together to achieve the effect that “1 + 1 is greater than 2” within the team, helping the team to generate value. In addition, the consistency of “Personnel Allocation” is 0.772727, which was the closest to the criterion for the necessary condition among the remaining conditional variables. This indicates that, although personnel allocation is not a necessary condition, it still has a significant impact on the perceived effectiveness of the PHCT.

Among the six path configurations, the “clear goals” dimension is the most direct and powerful driving force for PHCT effectiveness as it is required to participate in all paths. Second, “Personnel Allocation” appears in three path configurations, indicating that adequate personnel are a significant component of the process of providing complete PHC services. Research suggests that primary care physician–patient interaction time can be improved by enhancing the role of medical assistants as a team-based service model [41]. This phenomenon may be due to the fact that PHC services, as a systemic service process, require human resource support in all aspects. Increasing the allocation of team members can fully mobilize the team's work and promote continuous improvement of team effectiveness.

In addition, we were surprised to find that the effect of “Team Cohesiveness” was minimal in both the univariate necessity analysis and the multifactorial configuration analysis. This reflects the fact that team cohesiveness, as an intangible spiritual force within the PHC, plays a minor role in influencing team effectiveness. This is contrary to previous studies that found that good cohesion improves the efficiency and performance of team operations [42]. This may be because team members are less demanding in terms of an abstract spiritual core than figurative team goals and personnel allocation. Team members will be more inclined to comply with the rules and regulations that exist concretely in the organization and to fulfill their established responsibilities in the team in order to achieve the desired team effectiveness goals.

Two of the six path configurations are more typical of the path configurations. The path A accounts for approximately 45% of casesand explains how a team with adequate staffing, members with diverse disciplinary backgrounds, and clear team goals can form a combination of path configurations with a high level of PHCT effectiveness. This finding suggests that attention should be paid to the allocation of human resources to PHCTs. The services provided by the PHCT involve multiple tasks throughout the patient treatment process, and the entire process requires the allocation of an adequate number of health professionals in the team. The number of health human resources in the team ensures that PHC services are delivered smoothly to the population. Rational allocation of team health human resources can determine the quality of PHC service delivery. Therefore, scientific and reasonable planning of internal health human resources by team management is a powerful means of optimizing PHCT effectiveness. At the same time, organic coordination between the individual creativity of team members and that of the whole team should also be considered.

The pathway B suggests that even when personnel allocation, team cohesiveness, conflict management and resolution, and communication and information sharing elements are not present, but the team has clear goals, the effectiveness of PHCT can still be developed to some extent. Team members work individually around assigned goals, allowing the team to achieve efficient and high-level output. This confirms the direct impact of the “Clear Goals” element on the effectiveness of PHCTs. It prompts managers to pay attention to goal orientation. This is because people can hold themselves accountable with clear goals. Team managers should manage team members through setting goals. After the top management of the team determines the team goals, they are effectively decomposed and clearly transformed into sub-goals for each member of the team. Team members are evaluated according to the completion of sub-objectives. The clarity of team goals plays a positive role in guiding the work direction of team members and establishing division of labor and cooperation among team members. It is a clear catalyst for improving managerial efficiency and team effectiveness.

### Limitations and future studies

This study has some limitations. First, the design of the conditional variables inevitably carries a certain degree of subjective selectivity and limited horizon reviews by the researchers. Therefore, other important conditional variables that were not included in this study may exist. Second, pathways with a raw coverage of less than 10% were not discussed in this study because of the limited number of samples. Although the number of samples in this study was within the optimal range for the QCA method, the sample content covered by the pathways can still be expanded by increasing the number of samples in subsequent studies, with the aim of providing more accurate evidence. Third, due to the presence of COVID-19, the study was conducted only in Hangzhou, where the authors are located. Hangzhou has made PHC the focus of China's health care reform and has achieved considerable success; therefore, the development of its PHCT has a certain typicality. In less-developed areas, factors influencing the effectiveness of PHCTs may differ. In future studies, the expansion of the geographic scope and addition of samples from multiple regions could be considered to improve the universality of the study.

## Conclusion

Our analysis of PHCT members' perspectives on team effectiveness was based on their own experiences working as a team. On account of our interviews with PHCT members, we concluded that team members perceived that having a clear goal was the most immediate and powerful driver of PHCT effectiveness in the formation of team effectiveness, despite being in different PHCTs. Moreover, the improvement in PHCT effectiveness is not the result of a single factor; many factors jointly act to form a complex combination of conditions that affect team effectiveness. The next step is to share these findings with health authorities and primary care administrators to provide them with a basis for developing policies to improve PHCT effectiveness.

### Supplementary Information

**Additional file 1. **Interview outline.

**Additional file 2. **Results of software analysis of intermediate solution.

## Data Availability

The datasets used and/or analyzed during the current study are included in this manuscript. For any further data, it can be accessible from corresponding author in reasonable request.

## Abbreviations

PHC: Primary health care
PHCT: Primary health care team
TBC: Team-based care
QCA: Qualitative comparative analysis
csQCA: Crisp set qualitative comparative analysis

## References

[1] World Health Organization. Global status report on noncommunicable diseases 2014: attaining the nine global noncommunicable diseases targets;a shared responsibility. [R/OL]. (2014-01) [2023-08-30]. http://apps.who.int/iris/bitstream/handle/10665/148114/9789241564854_eng.pdf.

[2] Yaoyong B, Chaonian L (2016). Epidemic characteristics of hypertension and prevention strategies in China. J Diseases Monitor Control.

[3] Ruiming L, Qin C, Junhui X (2022). Constraints and optimization paths of policy implementation of family doctor contracting services in China: based on Smith’s policy implementation process model. Chin Gen Pract.

[4] Blain L, Flanagan PS, Shyr C (2021). Team-based care: a clinical pharmacist and family physicians. Can Pharmacists J / Revue des Pharmaciens Du Can.

[5] Lam Y, Daaleman T, Helton M (2018). Team-Based Care. Chronic illness care.

[6] Shasha Y, Fang W, Chenchen L (2014). Analysis of community health center general practice team composition model. Chin J Health Policy.

[7] Jin H (2020). Effectiveness evaluation and improvement strategy of family doctor team. Chin Gen Pract.

[8] Liqiang C, Jin H (2018). Study on the effect of "1 + 1 + 1" combination contracting on the effectiveness of family doctor services. Chinese General Practice.

[9] Fang W, Hongyue D, Guili C (2021). Study on the effectiveness and influencing factors of family doctor contracting service in Dongcheng District. Beijing. Chinese General Practice.

[10] Jiazhen Z, Bingyao M, Youqin S, et al. Analysis of the implementation effect and influencing factors of family doctor contract service system in Shenzhen. Chin J Hosp Adm. 2019;35(06) 447–51. Chinese.

[11] Gittell JH, Godfrey M, Thistlethwaite J (2013). Interprofessional collaborative practice and relational coordination: improving healthcare through relationships. J Interprof Care.

[12] Liu S, Wang L, Zhang T (2019). Factors affecting the work competency and stability of family doctors in Shanghai: a tracking study. BMC Fam Pract.

[13] Olga S, Torti Jacqueline MI, Kennett Sandra L, Bell NR (2018). Family physicians’ perspectives on interprofessional teamwork: findings from a qualitative study. J Interprof Care.

[14] Rowland. Core principles and values of effective team-based health care. J Interprof Care. 2014;28(1):79–80. 10.3109/13561820.2013.820906.

[15] Mukiapini S, Bresick G, Sayed AR, Le Grange C. Baseline measures of primary health care team functioning and overall primary health care performance at Du Noon Community Health Centre. Afr J Prim Health Care Family Med. 2018;10(1). 10.4102/phcfm.v10i1.1458. 10.4102/phcfm.v10i1.1458 PMC6131698 30198287

[16] Shortell SM, Marsteller JA, Lin M, Pearson ML, Wu SY, Mendel P, Cretin S, Rosen M (2004). The role of perceived team effectiveness in improving chronic illness care. Med Care.

[17] Song H, Chien AT, Fisher J (2015). Development and validation of the primary care team dynamics survey. Health Serv Res.

[18] Rize J, Hai F (2020). Research progress of family doctor contracting service in China based on supply and demand perspective. Chin Gen Pract.

[19] Xu Y, Juan C (2019). Analysis of the current service quality within a family doctor team in an urban area of Beijing. Chinese J Soc Med.

[20] Haoyang C, Shuoxiong F, Wenxi M (2022). Optimization study of family physician teams—based on team effectiveness model. Health Econ Res.

[21] Ragin CC. Redesigning Social inquiry. Chicago University P; 2008. 10.7208/chicago/9780226702797.001.0001.

[22] Short Kate E, Patricia, Kemp Lynn. Influential factor combinations leading to language outcomes following a home visiting intervention: a qualitative comparative analysis (QCA). Int J Lang Commun Disord. 2020;55(6). 10.1111/1460-6984.12573. 10.1111/1460-6984.12573 33051961

[23] Qingshun L, Lili L. A Qualitative Comparative Analysis of the Impact of Population Structure on the Growth of Medical Expenses. Popul Econ. 2020(5):103–17. (Chinese).

[24] Yi W (2020). Analysis of key influences on health levels in European Countries-A qualitative comparative analysis (QCA) based on 36 European countries. Chin J Health Policy.

[25] Yue L, Zhe Z, Huiyong Z (2011). Research on the theoretical framework of standard differentiation of TCM syndrome by using semi-structured interview method. Chinese Arch Traditl Chinese Med.

[26] Types of Health Care Quality Measures. Content last reviewed July 2015. Agency for Healthcare Research and Quality, Rockville, MD. https://www.ahrq.gov/talkingquality/measures/types.html.

[27] Rublee DA (1989). The quality of care: how can it be assessed?. JAMA.

[28] MyNvivo Portal. https://portal.mynvivo.com/shop/try-nvivo?plt=3.3.1.1.0=2.111209603.646055790.1651289991–595745408.1651289991. Accessed 22 Nov 2023.

[29] Rihoux B, Ragin CC. Configurational comparative methods: Qualitative Comparative Analysis (QCA) and related techniques. Sage. Retrieved from http://www.socsci.uci.edu/˜cragin/fsQCA/software.shtml.

[30] Goni S (1999). An analysis of the effectiveness of Spanish primary health care teams. Health Policy.

[31] Bower P (2003). Team structure, team climate and the quality of care in primary care: an observational study[J]. Qual Saf Health Care.

[32] Helfrich CD, Dolan ED, Fihn SD (2014). Association of medical home team-based care functions and perceived improvements in patient-centered care at VHA primary care clinics. Healthcare.

[33] Carey TA, Sirett D, Russell D (2018). What is the overall impact or effectiveness of visiting primary health care services in rural and remote communities in high-income countries? A systematic review. BMC Health Serv Res.

[34] Ragin C C, Davey S. 2009, fs/QCA: Fuzzy-set/qualitative comparative analysis. 2.5. Tucson: Department of Sociology, University of Arizona. Retrieved from http://www.socsci.uci.edu/˜cragin/fsQCA/software.shtml.

[35] Jiaojiao S, Yingzhi G, Yun Y. Why does China's urban tourism policy change? - Clear Set Qualitative Comparative Analysis based on Suzhou (csQCA) [J/OL]. Economic Geography:1–14[2022-07-07]. http://kns.cnki.net/kcms/detail/43.1126.K.20210108.1553.004.html. Chinese

[36] Jun W, Dianli W (2019). Research on the affecting factors of NIMBY conflict outcomes in China-based on 40 NIMBY conflicts cases through fsQCA. Journal of Public Management.

[37] Ragin CC, Sonnett J. Between Complexity and Parsimony: Limited Diversity, Counterfactual Cases, and Comparative Analysis. Vergleichen in Der Politikwissenschaft. 2005:180–197. 10.1007/978-3-322-80441-9_9.

[38] Yang H, Weiquan L, Xiongteng G (2019). Event properties, attention and policy agenda setting in the internet age: a qualitative comparative analysis based on 40 online focusing events. J Intell.

[39] Delva D, Jamieson M, Lemieux M (2008). Team effectiveness in academic primary health care teams. J Interprof Care.

[40] Sullivan EE, Ibrahim Z, Ellner AL (2016). Management lessons for high-functioning primary care teams. J Healthc Manag.

[41] Misra-Hebert AD, Rabovsky A, Yan C (2015). A team-based model of primary care delivery and physician-patient interaction. Am J Med.

[42] Xinlu R, Yongyong Z, Qingling Z (2016). Research on the team cohesion model of hospital knowledge workers: from the perspective of psychological contract. Chin J Geriatric Care.

