# Supplementary material for: The formation mechanism of primary health care team effectiveness : a qualitative comparative analysis research
Source: BMC Prim Care. 2024 Jan 29;25:45. doi: 10.1186/s12875-024-02278-8 (PMC10823627; doi:10.1186/s12875-024-02278-8)
Supplement: Supplementary file 1 — Additional file 1. Interview outline. [file 12875_2024_2278_MOESM1_ESM.doc]

Additional file 1 Interview outline

| **Interview outline** | |
| --- | --- |
| 1. | Are you currently serving as a team?  How many people do you have on your team?  Who is it made up of?  What is the division of labor within the team? (What are you mainly responsible for?) |
| 2. | How does your team provide services for patients?  In your opinion, what are the advantages and disadvantages of your team in providing services for patients? |
| 3. | How do you feel about your team's current service quality and efficiency?  What are the results of providing services to patients as a team?  Are you satisfied with your team and its service?  What factors do you think affect the quality and efficiency of your service? |
| 4. | Do you think there are currently factors affecting the effectiveness of your team's services?  What are the supporting factors? What are the barriers? |
| 5. | What other barriers do you think your team needs to break down in order to provide what you think is a better service for patients?  What improvements need to be made (such as capabilities or team composition)?  What conditions are needed for support (such as policy support, equipment support, etc.)? |
