# Supplementary figures and images for: The formation mechanism of primary health care team effectiveness : a qualitative comparative analysis research
Source: BMC Prim Care. 2024 Jan 29;25:45. doi: 10.1186/s12875-024-02278-8 (PMC10823627; doi:10.1186/s12875-024-02278-8)

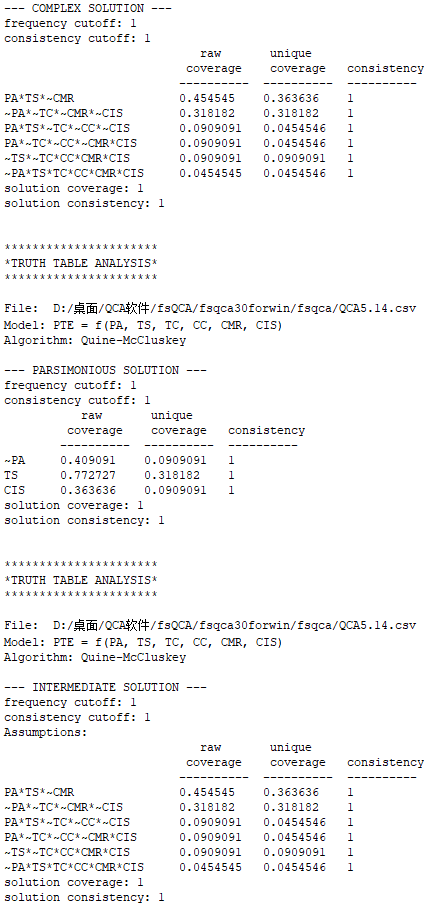


**Additional file 2 Results of software analysis of intermediate solution**

Supplement: Supplementary file 2 — Additional file 2. Results of software analysis of intermediate solution. [file 12875_2024_2278_MOESM2_ESM.doc]
